# *Leishmania* species in biopsies of patients with different clinical manifestations identified by high resolution melting and nested PCR in an Endemic district in Peru

**DOI:** 10.1016/j.parepi.2019.e00095

**Published:** 2019-02-11

**Authors:** Jesús Rojas-Jaimes, Nyshon Rojas-Palomino, James Pence, Andres G. Lescano

**Affiliations:** a Escuela de Medicina Humana, Universidad Científica del Sur, Lima, Peru; b Laboratorio de leishmaniasis, Instituto Nacional de Salud, Lima, Peru; c Facultad de Salud Pública y Administración, Universidad Peruana Cayetano Heredia, Lima, Peru

**Keywords:** *Leishmania*, Clinical manifestations, PCR, Genotyping, Cutaneous leishmaniasis

## Abstract

**Background:**

The parasite of the genus *Leishmania* causes a neglected disease known as leishmaniasis, which has different clinical aspects depending on the species that infects the person and the immune response of the patient. The objective was to determine, using molecular biology, the current circulating species of *Leishmania* in biopsies of different types of cutaneous leishmaniasis (CL) from the endemic district in the region of “Madre de Dios”, Peru.

**Methods:**

The study's 19 samples were obtained in the Huepethue district in the “Madre de Dios” region from patients who were diagnosed for suspected leishmaniasis infection with three clinical manifestations. These were analyzed using kDNA-PCR, Nested-PCR and HRM-PCR to identify the genus of an infecting parasite as well as its species.

**Results:**

kDNA-PCR detected all tests for the genus of *Leishmania* to be positive, while the Nested-PCR could not detect 20.9% of species (one *L*. (*V*.) *braziliensis* and three *L*. (*V*.) *amazonensis*), and the HRM-PCR detected all species presented in the samples. The most prevalent species was *L*. (*V*.) *braziliensis* (73.7%), and the clinical manifestations were ulcers (63.2%), superficial scabs (5.3%) and diffuse form (5.3%), followed by *L*. (*V*.) *amazonensis* (15.8%), manifesting ulcers (15.6%), and *L*. (*V*.) *lainsoni* (10.5%), manifesting superficial scabs (5.3%) and ulcers (5.3%). *L*. (*V*.) *braziliensis*, *L*. (*V*.) *amazonensis* and *L*. (*V*.) *lainsoni* were detected effectively using HRM-PCR in the samples with different clinical manifestations.

**Conclusions:**

Highlighting the findings of a high diversity of *Leishmania* species using High Resolution Melting PCR in biopsies with different clinical manifestations of cutaneous leishmaniasis.

## 1 Background

Cutaneous leishmaniasis is considered an emerging and uncontrolled disease by the World Health Organization and is characterized by a broad clinical presentation. The clinical manifestations are related among some *Leishmania* species. Additionally, some studies report a relationship between drug resistance developed during therapy and the *Leishmania* species, being L. *braziliensis* which is related to high drug resistance (Llanos-Cuentas et al., 2008). Therefore, there is clinical importance in identifying the *Leishmania* species in the patients who are in the endemic area. This study highlights the importance of comparing two molecular tools that use different molecular markers in order to identify the *Leishmania* species in an endemic area where different clinical manifestations of cutaneous leishmaniasis have been reported. Our findings help to select the best PCR to identify the *Leishmania* species in the clinical samples and increase the knowledge about circulating *Leishmania* species in a reduced area in Madre de Dios, where leishmaniasis is endemic.

Leishmaniasis a highly endemic disease that affects 17 different departments in Perú, is the second pandemic of a tropical nature, and is the third primary cause of death due to transmittable disease, following malaria and tuberculosis (Ministerio de Salud and Dirección General de Salud de las Personas, 1995; Saldaña et al., 2004; Alvar, 1996). Leishmaniasis is a parasitic infection caused by approximately 22 different species of protozoan hemoflagellates from the Leishmania genus, of which, only *L*. (*V*.) *peruviana*, *L*. (*V*.) *braziliensis*, *L*. (*V*.) *guyanensis*, *L* (*L*.) *mexicana*, *L*. (*V*.) *lainsoni*, *L*. (*L*.) *amazonensis and L*. (*L*.) *infantum* circulate throughout America. These parasites are transmitted through the bites of female mosquitoes, be they (from the *Phlebotomus* genus) – old world vectors or (from the *Lutzomyia genus*) – new world vectors, to rodents, canines, and other small mammals. In humans, new world infections can be separated into three clinical classifications, depending on their severity: Cutaneous Leishmaniasis (CL), Muco-cutaneous Leishmaniasis (MCL), and Visceral Leishmaniasis (VL) (Lee and Gilbert, 1999). This clinical form will depend on the infecting species and the response of immunological system of the patient (Lucas et al., 1998).

One of the first formal Peruvian studies (involving 15 departments of the country) focused on the molecular epidemiology of leishmaniasis and was able to link specific species (found in areas ranging from under 400 m AGL to 3000 m AGL) to clinical severity. The study showed that *L*. (*V*.) *braziliensis* was mainly responsible for the cutaneous localized and muco-cutaneous forms of the infection. In contrast, *L*. (*V*.) *guyanensis* (with some isolated cases showing a muco-cutaneous form), *L*. (*V*.) *peruviana*, *L*. (*V*.) *lainsoni*, *and L*. (*L*.) *amazonensis* were mainly responsible for cutaneous localized and diffuse forms of the infection in the case of L. *amazonensis*. In addition, *L*. (*V*.) *braziliensis* was found to be particularly common in the Madre de Dios region (Lucas et al., 1998; Tojal Da Silva et al., 2006).

One study, which focused on the historical characteristics of epidemiological incidents regarding leishmaniasis related to over infections and collateral effects of drugs, showed a high initial affliction rate with a relatively stationary period from 1950 to 1980, with a national mortality rate of approximately 6.55 to 8.45 people per 100,000. During 1984–1994, this rate increased to 12.69–40.02 people per 100,000 inhabitants, before showing a slight drop and stabilizing during the years 1994–1996 with a mortality rate of approximately 32.45 people per 100,000 inhabitants (Escalante, 1997). One of the zones with the highest rates of annual mortality is, as previously mentioned, Madre de Dios (Shender et al., 2014).

The most recent study regarding the aforementioned points dates back to 2010; it identified a new form of species that had appeared. This species has been denominated *L*. (*V*.) *shawi*, and was found close to the shared Peruvian/Brazilian border. This species was found with other prevalent species, including *L*.(*V*.) *peruviana*, *L*(*V*.) *brazilisensis and L*(*V*.) *guyanensis* (Kato et al., 2010).

These studies show the importance of identifying the different forms of leishmaniasis. Identification is also important because of a problem shown in another study, which investigated the relationship between the parasite and the lack of adequate therapeutic treatment. This research analyzed patients afflicted with *L*.(*V*.) *braziliensis*, *L*.(*V*.) *peruviana* and *L*.(*V*) *guyanenesis*; and it was determined that afflictions specifically caused by *L*.(*V*.) *braziliensis* were the most commonly misdiagnosed and the most poorly treated (followed by *L*.(*V*.) *peruviana* and *L*.(*V*.) *guyanensis*) (Llanos-Cuentas et al., 2008; Córdova et al., 2011; Foulet et al., 2007).

Identification is generally necessary in order to make a prognosis, particularly when referring to L*eishmania* (*V*.) *braziliensis*, the primary cause of chronic *Leishmaniasis*, which presents a unique ulcer like form with symmetrical scabbed edges and a tendency to evolve into the mucous variety of the infection. As for *Leishmania* (*L*.) *amazonensis*, if not treated appropriately, the normal cutaneous severity often becomes resistant to medication. This can also occur in *Leishmania* (*V*.) *lainsoni*, a species which, although rare, has been identified in Madre de Dios in a *Lutzomyia davisi* (Sanchez-Saldaña et al., 2004; Teles et al., 2015; Montalvo et al., 2012; Corrêa and Soares, 2006).

The present study aims to compare some molecular diagnosis of the parasite by identifying species of *Leishmania* sp. Using High Resolution Melting and Nested PCR in biopsies of patients with different clinical characteristics of cutaneous leishmaniasis.

## 2 Materials and methods

### 2.1 Study population

Our prospective study took place during May and June 2011, conducted with the intent of helping in the diagnosis of patients with different clinical manifestations of leishmaniasis cutaneous in the Huepetuhe district, located in an area where the informal population engages in illegal mining in the province of Manu, in the Madre de Dios region, Perú.

### 2.2 Ethics statement

After obtaining the informed consent of patients and an approval for conducting health-related studies issued by the Regional Health Directorate (DIRESA) of Madre de Dios' epidemiology area (institutional review board), 19 biopsy samples were collected with the permission of the patients.

All patients were adults or accompanied by an adult and their orally informed consent given was approved by the institutional review board. Informed consent was determined using two criteria: a) The refusal of acceptance of a document signed by a person working in illegal gold mining in Huepetuhe. The miners were working an illegal activity. Therefore, probably they did want to be identified. b) Information that the sampling was part of the support for the diagnosis and treatment provided free of charge by the national health system. The oral consent was documented by a treatment card that was filed by the health center.

### 2.3 Procedures

The inclusion of certain participants depended on two criteria: The first of these was prior clinical diagnosis of leishmaniasis, which was determined by a physician. Recognition of the disease was determined by the observation of some ulcerative wound with pronounced edges and slow deterioration. The characteristics of the disease are based on Peruvian technical standards (Ministerio de Salud and Dirección General de Salud de las Personas, 1995). In order to identify the geographic location of infection, the clinical evaluation included an epidemiological record in which the patient's stay in an endemic place is detailed at a minimum period of three weeks before the appearance of the ulcer, as well as the presentation of at least one positive result in the aforementioned tests as presence of amastigotes in the smear stained with Giemsa. Therefore, those excluded either presented no clinical diagnosis or tested negative in all clinical tests. The patients were enrolled passively in the district's main Reference Health Center, in the epidemiological survey the patients stated they had been residents of the Huepetuhe district approximately one month before and during the period of the disease**.**

The study used biopsy samples taken at the clinical laboratory in the health center in Huepetuhe, which were obtained by removing tissue from the infected regions. The area was first cleaned with 70% alcohol, after which it was injected with a local anesthetic (lidocaine 2%). A previously sterilized scalpel then cut along the external border of the skin lesion in order to obtain an approximately 3 mm tissue sample. These samples were catalogued and later divided. One piece of each sample was put through Giemsa staining and a 96% ethanol solution for later molecular testing. After a positive diagnosis by microscopy, the patients underwent treatment for CL (20 mg/kg/day of sodium stibogluconate intravenous for 20 days).

The first test sought to determine the presence of the parasite. A solution was prepared using 0.1 ml of leishmanine that contains 25–30 μg/mL of a *Leishmania* (*Viannia*) *peruviana* antigen. Using a syringe, this mixture was injected into the superior third of the anterior face of the forearm; the injection was superficial and was given 48 h to take effect. Any injection site, which showed swelling equal to or larger than 6 mm, was considered to test positive for the exposure of the parasite (Minaya et al., 1997).

The samples obtained were then imprinted on microscope slides and allowed to dry for 5 min after which, they were Giemsa stained for 15 min. These slides were then cleaned using water jetting and allowed to dry for 5 min. The slides were then observed at 1000× under a microscope in search for amastigotes.

### 2.4 kDNA-PCR to identify *Leishmania* genus

DNA was extracted using the Qiagen kit for tissues using the 3 mm of the samples approximately. For each PCR reaction 4 μl of DNA was used. In order to determine the presence of *Leishmania* (*V*) *braziliensis*, a positive control was used (LTB300 strain), as well as a negative control (this consisted of human DNA), and a target (distilled water, PCR grade in order to reveal cross-contamination).

The thermocycling conditions used in the GeneAmp® PCR System 9700, according to programing were: An initial denaturalization at 94 °C for 5 min followed by 35 cycles (denaturalization at 94 °C for 45 s, annealing of primers at 58 °C for 45 s, and extension at 72 °C for 1 min), with a final extension temperature of 72 °C for 5 min. This was then cooled to 4 °C and preserved at that temperature until use, to preserve the amplicon.

For the thermocycler machine, plastic stoppers were used as boundaries for a set of 0.2 ml PCR tubes. These tubes were later covered with a heated top before the reaction was allowed to run. The test took approximately 1:15 h. The primers used were -MP1-L (direct) 5′-TAC TCC TGC CCG ACA CTC TG-3 and MP3-H (reverse) 5′CGG GGT GAA TTC TGT ATG C-3′ with an expected product of approximately 70 pairs of bases (Lopez et al., 1993) (Lopez et al., 1993).

### 2.5 Nested-PCR in real time for the identification of *Leishmania* sp. species

The initial PCRs were performed using a Gene Amp PCR System 9700 thermocycler (Applied Biosystems) in a total volume of 50 μl containing 5 μl DNA, 1× PCR buffer (Invitrogen), 1 mM of each primer (Table 1), 1.5 U Platinum Taq DNA Polymerase (Invitrogen), 1.5 mM MgCl2, and 200 mM of each dNTP. Initial denaturation at 94 °C for 5 min was followed by 35 cycles of denaturation at 94 °C for 45 s, annealing at 57 °C (MPI- mannose phosphate isomerase) or 62 °C (6PGD-6-phosphogluconate dehydrogenase) for 45 s, and extension at 72 °C for 90 s; and a final extension at 72 °C for 7 min for MPI or 5 min for 6PGD.

**Table 1:** Test for the detection of the *Leishmania* sp. parasite.

	Percentage	Clinical manifestation	Smear	Leishmanine	kDNA-PCR	Nested real time-PCR	HRM-PCR
*L*. (*V*.) *braziliensis*	14(73.7%)	-Ulcer: 12(63.2%) -Superficial scab: 1(5.3%) -Diffuse: 1(5.3%)	-Positive: 13(68.5%) -Negative: 1(5.3%)	-Positive: 5(26.3%) -Negative: 2(10.6%) -ND: 7(36.9%)^a^	-Positive: 14(73.8%)	-Positive: 13(68.5%) -ND: 1(5.3%)	-Positive: 14(73.7%)
*L*. (*L*.) *amazonensis*	3(15.8%)	-Ulcer: 3(15.6%)	-Positive: 3(15.6%)	-ND: 3(15.6%)	-Positive: 3(15.6%)	-ND: 3(15.6%)	-Positive: 3(15.8%)
*L*. (*V*.) *lainsoni*	2(10.5%)	-Superficial scab: 1(5.3%) -Ulcer: 1(5.3%)	-Positive: 2(10.6)	-Negative: 1(5.3%) -ND: 1(5.3%)	-Positive: 2(10.6%)	-Positive: 2(10.6%)	-Positive: 2(10.5%)
Total	19(100%)	-Ulcer: 84.1% - Superficial scab: 10.6% -Difusse: 5.3%	-Positive: 94.7% -Negative: 5.3%	-Positive: 26.3% -Negative: 15.9% -ND:57.8%	-Positive: 100%	-Positive: 79.1% -ND: 20.9%	-Positive: 100%

a ND: Not determined.

In the Nested PCR independent reactions were developed for the MPI and 6PGD genes in the LightCycler 480 (Roche Applied Science) thermal cycler. 5 μl DNA of the first products was used for the Nested PCR. The Nested PCR reactions involved 1× LightCycler 480 Genotyping Master (Roche) containing a total volume of 20 μl, 1.25 mm of direct primer, 0.25 mm of a reverse primer, a 0.18 mm probe, a 0.18 mm probe sensor, and 5 mL of DNA (reference species were used as positive controls) otherwise known as PCR products.

The primers first round used were: (5! - 3!):

MPI MPI.ext.F CCC TTT GGT TGT CGG T.

MPI.ext.R TCA TAC GCA TAG GAG CA.

6PGD 6PGD. 909.F CAA GGC GTT CCC TAC ATT C.

6PGD. 1537.R TTG CGG TCG GGA CAA CTG G.

The primers used in the second round were: (5! - 3!):

MPI MPI.1082.F ACG CCC AAG TGG AAG GAT G.

MPI.1082.R ACA CCA CTG TAC CGT TCA CC.

MPI.1082. probe TTC CAG ACA GAA GCC CAG CCC AAT CGT CGG –.

MPI.1082.sensor red670 – GTC ACG GAG GTC GTC CCG CTT CCA G.

6PGD 6PGD.1262.F CAA GGA GAT GAA GGA GGG TC.

6PGD.1262.R CTT GTC AAC ACG TTC GTA GC.

6PGD.1262. probe GCC AGG GAG GCA GTC ATC ACC G –.

6PGD.1262.sensor red610 – AAC GAT ACA GCC GTG CTC GC (Tsukayama et al., 2013).

The PCR consisted of an initial denaturalization at 95 °C for 5 min, followed by 45 cycles of denaturalization at 95 °C for 10 s, an alignment time of 20 s at 60 °C, and an extension period at 72 °C for 20 s.

The recommendations in the theoretical guide were followed to avoid cross-contamination, including physical separations of PCR reactions and their amplified products, the use of ultraviolet light to remove traces of DNA on work surfaces, and the application of alicuota to the reagents and master mix.

For the tests, 1 ng of *L*. (*V*) *braziliensis* DNA was used as the positive control and a sample of nuclease-free water as a negative control. For the species identification reactions, 1 ng of DNA from *L*. (*V*.) *braziliensis*, *L*. (*V*.) *peruviana*, *L*. (*V*.) *guyanensis*, *L*. (*V*.) *panamensis*, *L*. (*L*.) *lainsoni*, *L*. (*L*.) *amazonensis*, and *L*. (*L*.) *mexicana* were used. The DNA also functioned as positive/negative controls.

### 2.6 Identification of species of *Leishmania* using high resolution of disassociation PCR “high resolution melting”

The purified DNA of the biopsy was then tested in real-time in the HRM-PCR with a concentration of 5 ng/μl of DNA and 0.7 μm of the following primers OL (Origin of replication of the minicircle molecule *Leismania* kinetoplast): OL1 - Direct 5′GGG GAG GGG CGT TCT GCG AA 3′ and OL2 - Reverse 5′CCG CCC CTA TTT TAC ACC AAC CCC 3′. The fluorophore Eva Green (Tipe-itHRMPCRkit; Qiagen) was used for 35 cycles consisting of: denaturalization at 95 °C for 10 s, ring-formation at 55 °C for 30 s, and an extension period at 72 °C for 10 s. This was followed by a 1 °C progressive increase of temperature in the range of 75 °C to 95 °C in order to achieve the denaturalization of the double strand of the amplicon and the dissociation of the fluorophore (Rojas-Palomino et al., 2013).

The analysis of the dissociation curves for species differentiation of Leishmania sp. was performed with a 90% confidence interval using the software Rotor Gene Q version V2.1.0.

### 2.7 Statistical methods

A double-entry table was used to relate the *Leishmania* species to the clinical manifestations, along with the microscopic and molecular techniques.

## 3 Results

### 3.1 Probable infection sites

**Fig. 1:**
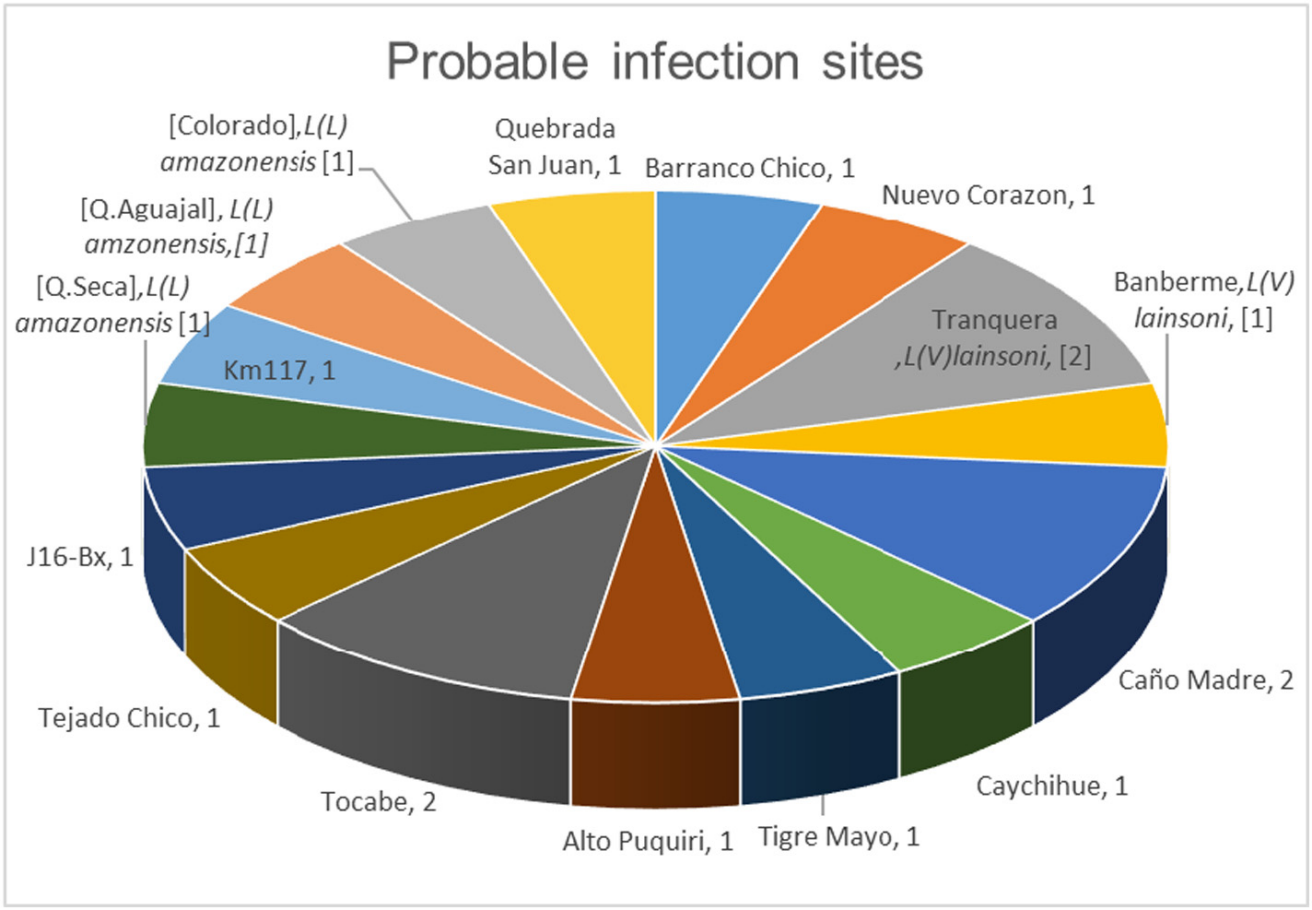
Probable infection sites. *Only *L*. (*L*) *amazonensis* and *L*. (*V*) *lainsoni* are specifically written on the graph, another infection was caused by *L*. (*V*) *braziliensis*. Size in the area in the chart depend of the number of the infected patient. *J16-Bx 1: A code representing biopsies whose origins of the infection could not be identified.

**Fig. 2:**
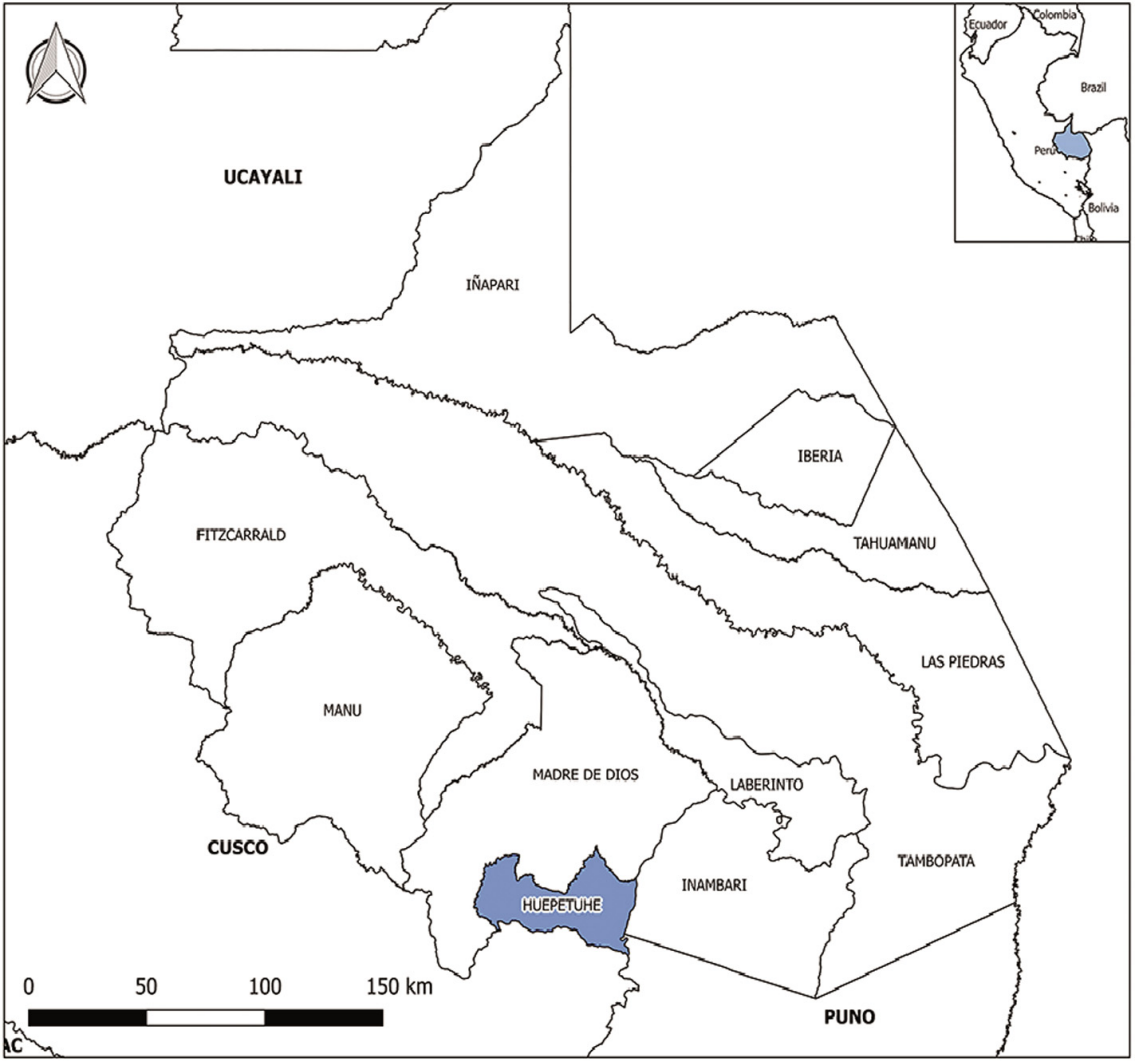
Map of the zone study.

Sites such as Tocabe, Caño Madre, and Tranquera have the highest infection rate (Fig. 1), keeping in mind that all other areas only show one person infected per area. It is also important to state that probable sites of infection include all areas where illegal mining exists. The activities of illegal mining sites were reported by the patients and corroborated by health personnel residents of the Huepetuhe district, Madre de Dios, Peru (Fig. 2).

### 3.2 Age and gender

94.8% of patients infected with *Leishmania* sp. parasites were male and the ages varied between 13 and 56 years old.

### 3.3 Diagnostic tests

Amastigotes were visualized in 18 (94.7%) of the biopsy samples, with one biopsy determined to be a false negative, due to Giemsa staining. 3/8 (37.5%) of the patients tested negative for leishmanin. Not all patients received leishmanin because of the lack of the reagents. In the molecular tests, the kDNA-PCR was able to detect *Leishmania* sp. genus in all the biopsy samples, the Nested qPCR could not detect 20.9% of species, and the HRM-PCR detected all of the species in the samples. In the case of HRM-PCR only the areas with a confidence of similarity greater than 90% compared to the area of the reference strain were defined as genotyped samples (Table 1).

## 4 Discussion

Epidemiological study is key in the control of leishmaniasis (Rodríguez et al., 1985; Osores et al., 2012). The findings, with the support of molecular biology, showed that kDNA-PCR, a test widely used for its sensitivity and specificity, tested positive for all samples analyzed in the detection of the genus *Leishmania* sp. (Lopez et al., 1993). As for the identification of species, the Nested RT-PCR described by Tsukayama (which used parasitic genomic DNA) was limited, as not all species could be identified due to a limitation in the sensitivity (Tsukayama et al., 2013). In contrast, the HRM – PCR a real time PCR identified all species of *Leishmania* sp. HRM-PCR uses the DNA of the mini-circle in the kinetoplast, which can have thousands of copies in a single parasite, thus improving the sensitivity of the test as well as allowing the differentiation of the subgenus *Viannia* and *Leishmania* in a single reaction (Rojas-Palomino et al., 2013). This, consequently, saves time in the identification of the parasite.

The molecular identification of species in our study showed that the most prevalent was *L*. (*V*) *braziliensis*, with a prevalence of 73.8%. This data is of particular epidemiological importance because the species itself is one of the few parasites to evolve a mucosal form from its original ulcerative (cutaneous) form, and is one of the only leishmania species to do so in all Latin America. This also makes treatment difficult, implicating the need for second cycles of antiparasitics that attack the disease in a state of remission (Teles et al., 2015). Additionally, it is worth noting that the clinical forms of *L*. (*V*.) *braziliensis* that were found in three different patterns displayed a superficial scab-like appearance with irregular borders. The diffuse form, as well as an ulcerative case showed a disseminated nodular inflammation with atypical lesions and the classical ulcer. These different clinical manifestations result from the implication of the interaction between the parasite and the host.

In the case of *L*. (*L*) *amazonensis*, it maintained a presence of 15.6% in testing, making it the second most common in this study. A high frequency of *Leishmania amazonensis* was found as national reference. That found in our study is interesting because no other reports in Peru document similar or greater frequencies of *L*. (*L*) *amazonensis* than we reported. In previous studies, the species was isolated after a therapeutic failure as well as a failure in the Th1 type cellular response. This often provokes a form of parasitic metastasis and in some cases a diffusion of the leishmaniasis parasite (Teles et al., 2015; Teles et al., 2016). This contrasted with our study, as the clinical forms were ulcerative with a single lesion.

In the case of *L*. (*V*) *lainsoni*, a clinical development was reported in 2010 in Madre de Dios, near the border with Brazil (Tejada, 1973). Its presence was also noticed in our study as two clinical samples (conforming a “10.6%” of the total) tested positive for the species. One of the hosts presented an atypical crusting scab that presented irregular edges and another presented the classical ulcerative case.

In the case of microscopy, the presence of amastigotes was found in nearly all samples excluding one, which was later found to be a false negative. In the case of leishmanina, a high specificity test used in probable cases of leishmaniasis resulted in three false negatives (possibly due to immunological cellular anergy modulated by the parasite) (Montalvo et al., 2012).

It should be noted that the present study is the first report from the district of Huepetuhe (an area of 1478 km^2^ with a population of 6495 inhabitants), where 80% of the gold-mining activity in Madre de Dios is concentrated. It is in the middle zone adjacent to the Cusco region (Rojas-Palomino et al., 2013). Regarding gender, 94.8% are males and 5.2% are females. Of these, 15.79% of people between the ages of 10 to 19 and 84.21% of people from 20 to 56 are infected. These results could be linked to the high index of mining and logging activity as well as the high percentile of males in adulthood (Rodríguez et al., 1985). It is important to mention that Huepetuhe is located in the jungle area of Madre de Dios, one of the places with the most biodiversity in the world. However, this place is one of the oldest settlements dedicated to illegal mining in Madre de Dios, where infectious diseases are more frequent because of prostitution, poor water quality, exposure to mercury, and the migration of people working in mining, increasing the possibility of transmission of pathogens by vectors because of poor health conditions due to malnutrition, infectious diseases and exposure to heavy metals in the population. Additionally, is important to mention that miners were exposed to vector bites when entering the jungle (Rojas-Palomino et al., 2013; Guthmann et al., 1997).

A key limitation of our study was the absence of a larger range of samples as well as the absence of a larger study area.

## 5 Conclusion

The study notes that an important molecular diversity of species (15.80%) was identified using HRM-PCR. Even though the study was carried out in a small number of patients. *L*. (*V*) *braziliensis*, *L*. (*L*) *amazonensis*, and *L* (*V*) *lainsoni* (from highest to lowest prevelance) were identified, noting that the pathogens found were identified in patients with different types of cutaneous leishmaniasis residing in the smallest endemic district of Madre de Dios. All evidence suggests that High Resolution Melting PCR is better than Nested PCR because the number of copies amplified with HRM is greater than number of copies in the Nested PCR.
